# Sex differences in the Kimchi-Palmer task revisited: Global reaction times, but not number of global choices differ between adult men and women

**DOI:** 10.1016/j.physbeh.2016.07.012

**Published:** 2016-07-18

**Authors:** Andrea Scheuringer, Belinda Pletzer

**Affiliations:** a Department of Psychology, University of Salzburg, Salzburg, Austria; b Centre for Cognitive Neuroscience, University of Salzburg, Salzburg, Austria

**Keywords:** Kimchi-Palmer task, Global-local processing, Sex differences, Menstrual cycle, Sex hormones, Mood

## Abstract

Research, directly assessing sex-dependent differences in global versus local processing is sparse, but predominantly suggesting that men show a stronger global processing bias than women. Utilizing the Kimchi-Palmer task however, sex differences in the number of global choices can only be found in children, but not in adults. In the current study 52 men and 46 women completed a computerized version of the Kimchi Palmer task, in order to investigate whether sex-differences in global-local processing in the Kimchi-Palmer task are reflected in choice reaction times rather than choices per se. While no sex differences were found in the number of global choices, we found that especially women are faster in making local choices than men, while men are faster in making global choices than women. We did not find support for the assumption that this sex difference was modulated by menstrual cycle phase of women, since the difference between reaction times to global and local choices was consistent across the menstrual cycle of women. Accordingly there was no relationship between progesterone and global-local processing in the Kimchi-Palmer task. However, like in studies utilizing the Navon task, testosterone was positively related to the number of global choices in both men and women. To our knowledge, this is the first study including reaction times as outcome measure in a Kimchi Palmer paradigm and also the first study demonstrating sex differences in the Kimchi Palmer task in adults.

## 1 Introduction

Most stimuli in everyday life are of a hierarchical nature, i.e. a global or higher order figure is composed of several local and more detailed features. People can either process scenes and stimuli in a global way, by attending to the figure as a whole or in a local way, by processing the details of a scene or the single parts of a figure.

Global and local visual processing has traditionally been assessed with a Navon task [1], in which participants are presented hierarchical stimuli. Large letters or shapes (global level) are composed of smaller letters or shapes (local level), which are evenly distributed in the larger letter or shape. Participants are asked to respond if they detect a target stimulus at the global or local level, respectively. Navon [1] demonstrated overall faster responses to the global, compared to the local level.

There are many factors influencing global-local processing in the Navon task, like the stimulus category (shapes or letters), stimulus properties or visual angle of presentation [2–6]. Research also indicates that mood and sex relate to global-local processing in a Navon task. Regarding mood, positive affect has been linked to a global processing bias, i.e. faster global responses, while negative affect has been linked to a local processing bias, i.e. faster local responses [7–11]. Regarding sex differences, increased global processing bias, i.e. faster responses to global targets were observed in men compared to women ([12–15], but see also [16]). Most recent results suggest that these sex differences are driven by opposite relationships of testosterone and progesterone to the global processing bias (i.e. faster responses to global stimuli) and may thus be restricted to the luteal cycle phase in women, when progesterone levels peak [14]. Interactive effects of sex and mood on global-local processing have however not been previously investigated, even though sex and menstrual cycle effects on mood have repeatedly been demonstrated [17–19].

**Fig. 1:**
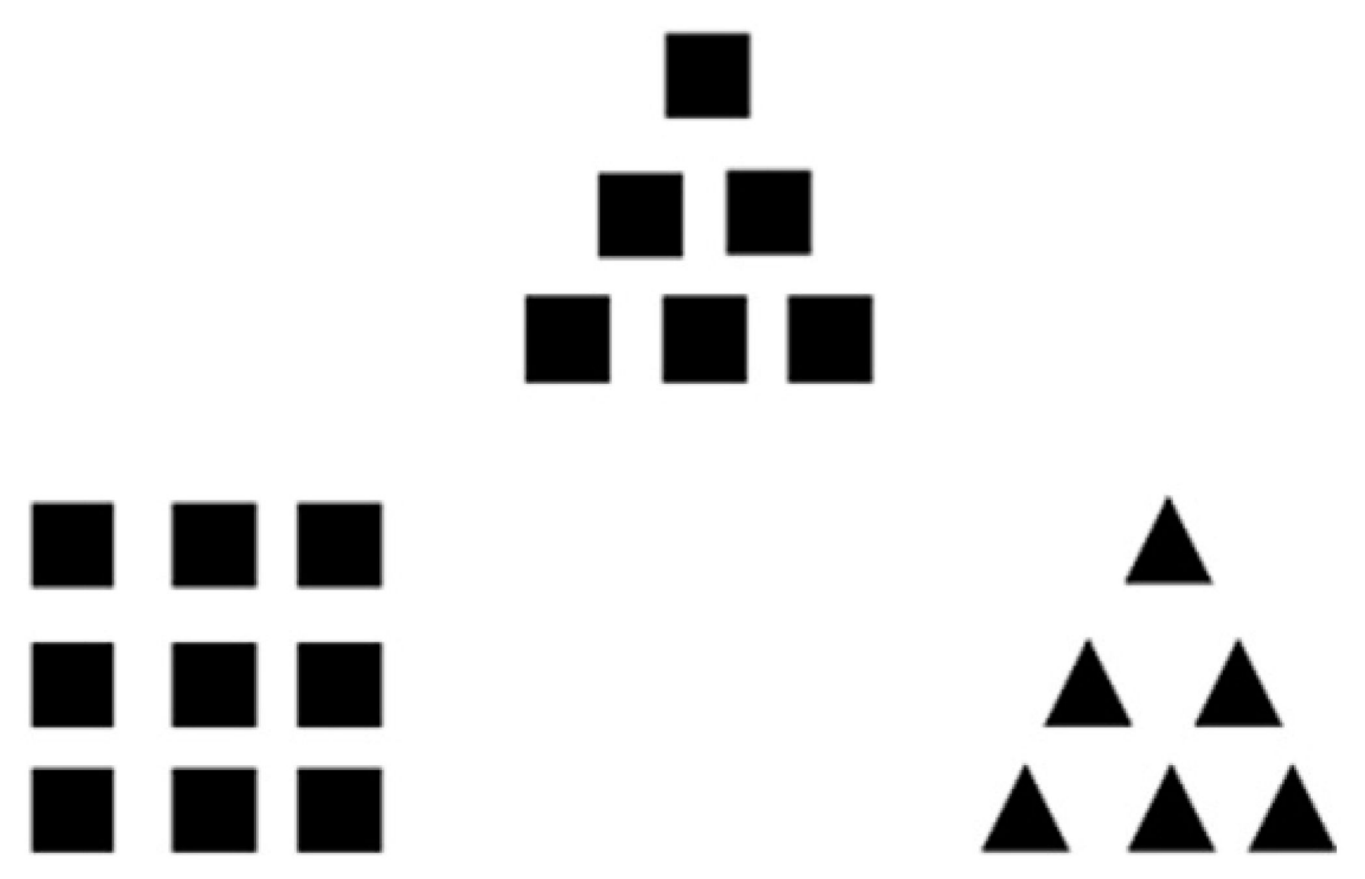
An example stimulus of the Kimchi Palmer task. The figure on the top is the target figure. Participants were asked to decide, which of the two figures, presented at the bottom, is subjectively more similar to the target figure.

Similar in stimulus architecture to the Navon task is the Kimchi Palmer task [20]. Like Navon stimuli, Kimchi Palmer stimuli consist of a global shape (square or triangle) made up of local shapes (squares or triangles). Participants are presented with two figures, which should be compared against a target or standard stimulus on the basis of subjective perceptual similarity. One of the figures matches the target stimulus at the global level (global match), the other one at the local level (local match). For an example stimulus, see Fig. 1.

Research with children, using the Kimchi Palmer task revealed large and reliable sex differences in the number of choices for global versus local matches [21–22]. They observed an increased number of global choices in boys compared to girls. Age of children in these studies ranged between 4 and 12 years [21] and 6 to 7 years [22]. On the contrary, Basso and Lowery [23] were not able to detect sex differences regarding the number of global and local choices in adults. Thus, sex difference research with the Navon task revealed significant sex differences in adults, whereas significant sex differences in the Kimchi Palmer task were only found in children.

Both tasks have been introduced as measures of global-local processing [1; 20]. However, while they are similar in stimulus architecture, there are several characteristics that distinguish between the Navon task and the Kimchi Palmer task.

First, the Navon task is usually implemented as a target detection paradigm, while the Kimchi Palmer task is implemented as a similarity judgment paradigm. Instead of searching for a predefined target, participants are asked to decide which match is more similar to the target stimulus. Note however that some studies also implement the Navon task as a similarity judgment paradigm and confirm faster responses for global matches than for local matches [24].

Second, in the Navon task participants are explained what the global and the local level are and are asked to detect targets or rate similarity for a predefined level (global or local). Thus, in the Navon task there is an objectively correct answer to each item. In the Kimchi-Palmer task however, participants are unaware of the distinction between global and local level and are specifically instructed to base their choices on subjective similarity.

Third, the Navon paradigm is computerized and participant's response times are restricted, requiring quick and spontaneous responses. Thus, the primary outcome measure in the Navon task is the reaction time. On the contrary, the primary outcome measure in the Kimchi-Palmer task is participant's choice. The Kimchi Palmer task is traditionally presented in a paper-and pencil format without a predefined time limit. While participants are instructed to respond as quickly as possible, they can choose on their own how long they work on each item. Thus responses in the Kimchi-Palmer task may be less spontaneous than in the Navon task, which may present an issue in studies on adults.

Therefore, the present study aims to address whether response times also represent a useful outcome measure in the Kimchi-Palmer task. Therefore, in the present study, we present the traditional Kimchi-Palmer stimuli on a computer screen, where participants had the possibility to choose between global and local matches and record the response times for participant's choices. All other aspects of the task are kept as in the traditional format, i.e. there was no time limit for participant's choices and they were not instructed about the global and local level.

We aim to investigate, whether sex differences in global-local processing during the Kimchi-Palmer task in adults, may not be reflected so much in their choices, but in the time they take to make these choices, i.e. their response times. We hypothesize that decisions for global matches are faster than decisions for local matches in men, while decisions for local matches are faster than decisions for global matches in women. Based on recent results from the Navon paradigm [14], we furthermore examine if fluctuations in sex hormones and female cycle phase relate to participant's performance in the Kimchi-Palmer task. More specifically, we assume that women show an increased global processing bias (i.e. faster responses to global matches than local matches) in the follicular cycle phase, compared to their luteal phase. We further hypothesize that global choices are more frequent and faster, the higher the testosterone levels, whereas local choices are more frequent and faster, the higher the progesterone levels. Furthermore we address, whether fluctuations in mood are associated with potential changes in global-local processing across the menstrual cycle.

## 2 Method

### 2.1 Participants

104 (53 men, 51 women) German-speaking participants completed the present study. Five women had to be excluded from analyses because of inconsistencies between the self-reported cycle phases and the analyzed hormone values (see below). Two men were excluded because testosterone levels varied highly between the two test sessions, indicating external factors influencing testosterone levels, like stress. Thus a total of 7 participants were excluded prior to analyses because of inconsistencies in hormone values and all analyses were performed on data of 51 men (*M*_age_ = 23.59, SD = 4.07) and 46 women (*M*_age_ = 22.80; *SD* = 3.47)

Age of all participants ranged between 18 and 36 and did not differ significantly between men and women (*t*_(98)_ = -1.02, *p* = 0.31). The majority of participants were students of the University of Salzburg, who received course credits for participation. All participants gave their informed written consent to participate in the study and all methods conform to the Code of Ethics of the World Medical Association (Declaration of Helsinki).

Only participants who reported to be right-handers and had no neurological, psychological or endocrinological disorders were included in the study. Women were only included in the study, if they reported no use of hormonal contraceptives and a regular menstrual cycle of a duration between 21 and 36 days [25]. The mean cycle length of women in the present study was 29.17 days (*SD* = 2.56). All participants were tested twice. While men participated within an interval of about 2 weeks, women participated in the study in two different cycle phases, i.e. the early follicular phase (days 1–6 of their menstrual cycle) and the mid-luteal phase (3–10 days after ovulation). Ovulation was calculated based on participants self-reports and confirmed by commercial ovulation tests. 21 women were in their follicular cycle phase during the first session and in their luteal cycle phase during the second session. 25 women were in their luteal cycle phase in the first session and in their follicular phase in the second session, respectively. 5 (2 men, 3 women) participants did not return for the second test session. Their data from the first test session were included in the analyses.

### 2.2 Kimchi Palmer task

As part of a larger study, participants completed the Kimchi-Palmer task [20], where they had to make subjective similarity judgements using hierarchical figures. A triad of large/global triangles or squares composed of small/local triangles or squares (for an example see Fig. 1) was presented on a computer screen using Presentation Software, Version 18.0 (2014, Neurobehavioral Systems Inc., Albany, CA, USA).

For each stimulus there was one target figure on the top and two comparison figures on the bottom. The target figure was always a square made up of triangles or a triangle made up of squares. One of these comparison figures matched the global level of the target stimulus, but consisted of different local elements (same-configuration comparison pattern), the other comparison figure matched the target stimulus on the local level, i.e. had the same elements, but a different global pattern (same-element comparison pattern). Participants' task was to decide, which comparison figure in their opinion matched the target figure more closely. They were instructed to decide for one of the two comparison figures on the bottom as quickly as possible and that there was no correct or incorrect decision. They responded with the left mouse key, when deciding for the comparison figure on the left side and with the right mouse key, when deciding for the comparison figure on the right side. Although, participants were instructed to respond as quickly as possible, they did not have a specific prescribed time limit. Completing this task lasted about 3 to 5 min.

The original Kimchi-Palmer item set consists of 16 low-density items with 3 or 4 local elements per global figure and 8 high-density items with 6–9 local elements per global figure. 12 of the 24 items had their global comparison figure (same-configuration comparison pattern) on the left side, whereas the other 12 items had their global comparison figure on the right side. In 12 items the global match was a triangle, in 12 items the global match was a square.

In order to control for memory effects two different versions of the Kimchi-Palmer task were created as follows. 8 items each were randomly assigned to one version of the task. The remaining 8 items were presented in both versions. Thus, each version consisted of a total of 16 items. In version A, 12 of the items were low-density and 4 high-density. In Version B, 10 items were low-density and 6 high-density. In each version, 8 items each had their global match on the right and left side respectively.

56 participants (29 women, 27 men) received Version A in the first session and Version B in the second session and 48 participants (22 women, 26 men) received Version B in the first session and version A in the second session, respectively. In order to assess global and local processing the percentage of global and local choices, as well as reaction times for global and local choices were recorded for each participant. A linear mixed model with '*session*' and '*version*' as factors and participant's '*choice*' as dependent variable revealed that the two versions did not differ significantly in the number of global choices. A linear mixed model with '*choice*', '*session*' and '*version*' as factors and participants 'reaction times' (RT) as dependent variable, revealed that the two versions did furthermore not differ in global or local RT.

### 2.3 Mood

In order to control for mood, we used a short questionnaire assessing positive and negative affect, the PANAS [26]. It examines the positive and negative affect of participants in their actual state. Every subscale contained 10 items, more precisely adjectives, describing affective states and participants should assess on a 5-point Likert-type scale (1 = “not at all” to 5 = “extremely”) how they felt at the moment. Example items were adjectives such as nervous, guilty, alert, irritable, hostile, and vigorous. For the current study the German version was used. The internal reliability varied between Cronbach's α = 0.85 and α = 0.86 for the subscale '*Positive Affect*' and between α = 0.74 and α = 0.86 for '*Negative Affect*', depending on the cycle phase or session.

### 2.4 Hormone levels

To examine whether sex hormones relate to performance in the Kimchi-Palmer task, we assessed testosterone, estradiol, and progesterone levels from saliva samples using ELISA kits from DeMediTec. Overall 3 saliva samples were taken from each participant during each session, and hormone levels were averaged over the three samples in order to control for diurnal fluctuations in hormone levels and to ensure reliability of hormone assessment. Until hormone assessment, saliva samples were stored at - 20 °C and centrifuged at 3000 rpm for 20 min.

### 2.5 Statistical analyses

36 responses to individual items with response times higher than 3 standard deviations over the mean (*M* = 1810,17 ms; *SD* = 4359,61 ms; exclusion criterion > 14,889 ms) were identified as outliers and thus excluded from analyses, because extensively long response time may no longer reflect spontaneous decisions as instructed.

Statistical analyses were carried out in R 3.2.2. Linear mixed effects models were utilized using the lmer function of the lme4 package. Dependent variables are participants '*choice*' and 'RT'. All models control for repeated measurement by modulating participant number as random factor. For the dependent variable '*choice*', the fixed factors ‘*session*’, ‘*density*’ and ‘*sex*’ were modelled, in order to compare the number of global and local choices between men and women. In women only, a model including the fixed factors ‘*session*’, ‘*density*’ and ‘*cycle*’ was run, in order to assess menstrual cycle dependent effects. For the dependent variable RT, the fixed factors ‘*choice*’, ‘*session*’, ‘*density*’ and ‘*sex*’ were modelled, in order to investigate whether global choices were faster than local choices and whether this difference was affected by sex. In women only, a model including the fixed factors, ‘*choice*’, ‘*session*’, ‘*density*’ and ‘*cycle*’ was run, in order to assess menstrual cycle dependent effects on this difference. Later, '*progesterone*' and '*testosterone*' levels, as well as '*positive*' and '*negative*' affect were separately added as additional fixed effects to the models. Each model also included all possible interactions between fixed effects. Non-significant interactions were then backwards eliminated using the step function of the lmerTest package at its default settings to create minimum models including only the factors and interactions relevant for explaining the dependent variable. Results of these minimum models are reported. The step-function does approximate degrees of freedom via a Satterthwaite approximation.

## 3 Results

### 3.1 Hormone levels

Progesterone was significantly higher in women compared to men (*b* = - 161.02, *SE*_b_ = 41.81, *t*_(90)_ = -3.85, p < 0.001) and within women significantly higher during the luteal compared to the follicular phase (*b* = 212.38, *SE*_b_ = 42.48, *t*_(41)_= 5.00, *p* < 0.001). Testosterone was significantly higher in men compared to women (*b* = 96.77, *SE*_b_= 13.57, *t*_(91)_= 7.13, p < 0.001) and did not differ significantly between cycle phases in women (All means are displayed in Table 1).

### 3.2 Sex and menstrual cycle differences in the number of global choices

**Fig. 2:**
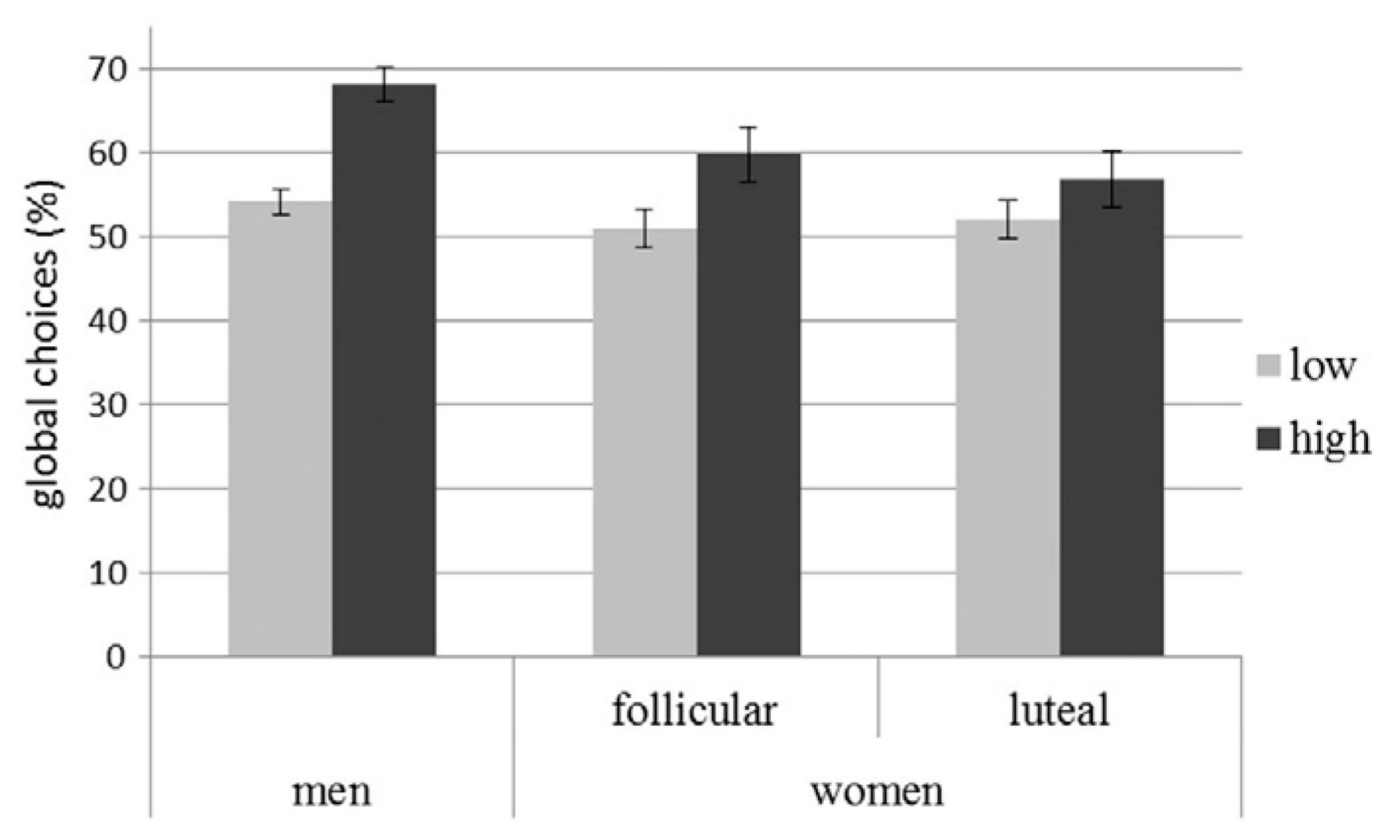
Percentage (%) of global with low vs. high density items in men and women. All participants made significantly more global choices with high-density items, compared to low density-items (p < 0.001).

In the total sample, neither ‘*session*’, nor ‘*sex*’ had a significant effect on the dependent variable ‘*choice*’.^1^ In women, neither ‘*session*’, nor ‘*cycle phase*’ had a significant effect on the dependent variable ‘*choice*’. Thus, only ‘*density*’ remained in the model (*b* = 0.11, *SE*_b_= 0.02, t_(2889)_= 6.53, p < 0.001). All participants made significantly more global choices with high-density items (64%) than with low-density items (53%), irrespective of ‘*sex*’ or ‘*session*’. In women, the effect of density did furthermore not depend on menstrual cycle phase (compare Fig. 2). Therefore, ‘*sex*’, ‘*session*’ and ‘*cycle phase*’ were not considered further in the analysis of choices.

### 3.3 Sex and menstrual cycle differences in reaction times associated with global vs. local choices

**Fig. 3:**
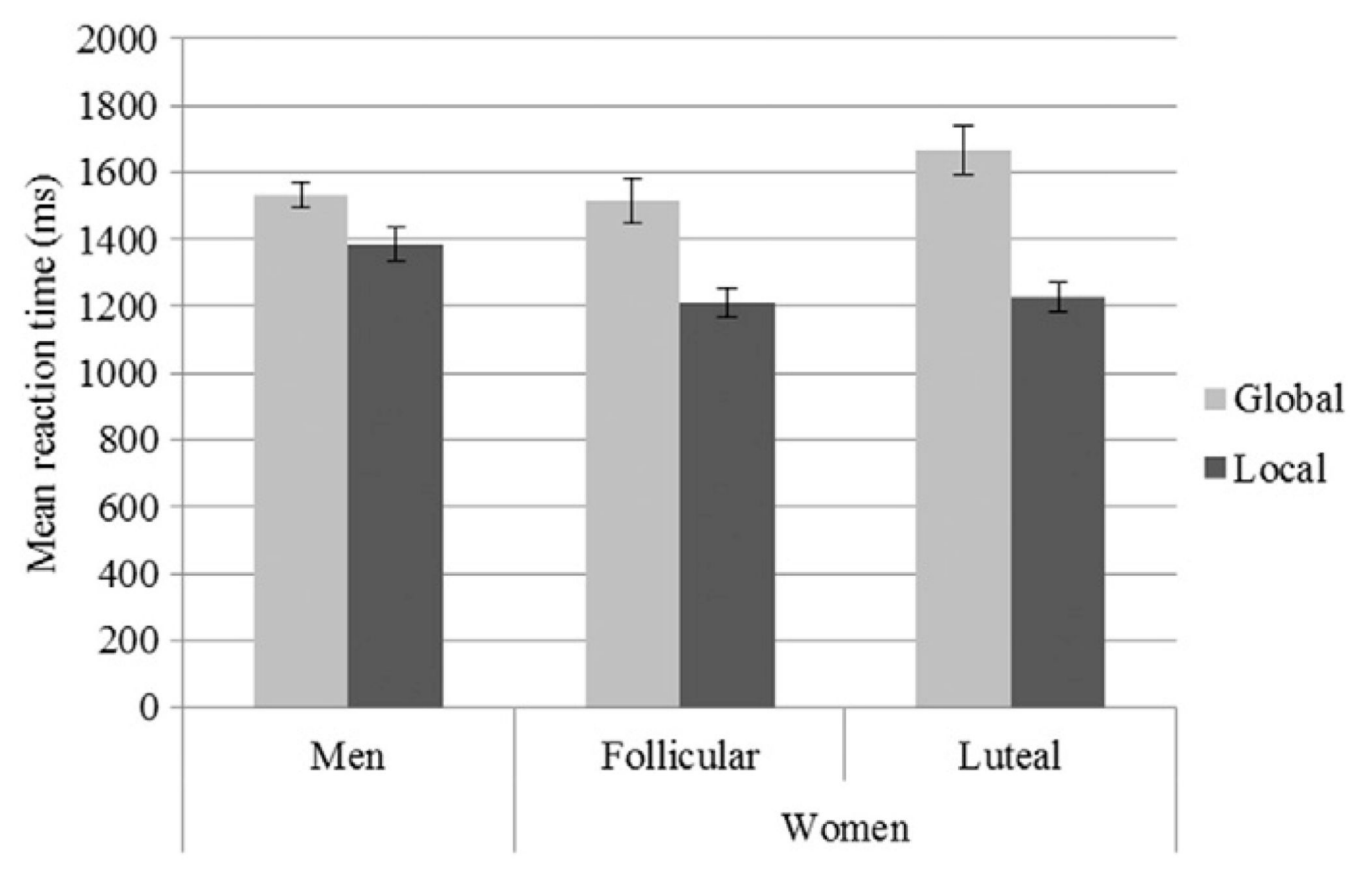
Mean reaction time (RT) for global and local choices in men and women. In all participants local choices were faster than global choices. Irrespective of cycle phase, local choices were significantly faster in women than in men, while global choices were faster in men than in women.

‘*Density*’ did not affect ‘*RT*’ and did also not interact with ‘*sex*’, ‘*choice*’ or ‘*session*’ and was therefore not considered further in the analysis of ‘*reaction times*’. Irrespective of ‘*sex*’ or ‘*choice*’, ‘*session*’ had a significant effect on ‘*RT*’ (*b* = -189.73, *SE*_b_= 39.92, *t*_(3771)_= -4.75, p b 0.001), with faster reactions during the second test session. There was a significant main effect of ‘*choice*’ on ‘*RT*’ (*b* = 349.71, *SE*_b_= 67.69, t_(3238)_= 5.16, p < 0.001), with global choices being slower than local choices. Thus, the larger the effect of ‘*choice*’ on ‘*RT*’, the faster are the local choices and the slower are the global choices, which is indicative of stronger local processing. Whereas, the smaller the effect of ‘*choice*’ on ‘*RT*’, the faster are the global choices and the slower are the local choices, which is indicative of stronger global processing. There was only a trend main effect of ‘*sex*’ (*b* = 173.41, *SE*_b_= 103.18, *t*_(160)_= 1.68, *p* = 0.09), indicating slightly slower reactions in men compared to women. A significant ‘*choice*’ * ‘*sex*’ interaction (b = -259.02, *SE*_b_= 90.92, *t*_(3486)_= -2.85, *p* = 0.004), indicated that the effect of ‘*choice*’ was significantly larger in women than in men. Local reactions were faster in women than in men, while global reactions were faster in men compared to women (compare Fig. 3). In women, ‘*menstrual cycle phase*’ did not affect ‘*RT*’ and did not interact with the effect of ‘*choice*’ on ‘*RT*’.

### 3.4 Sex hormone influences on the number of global choices

**Fig. 4:**
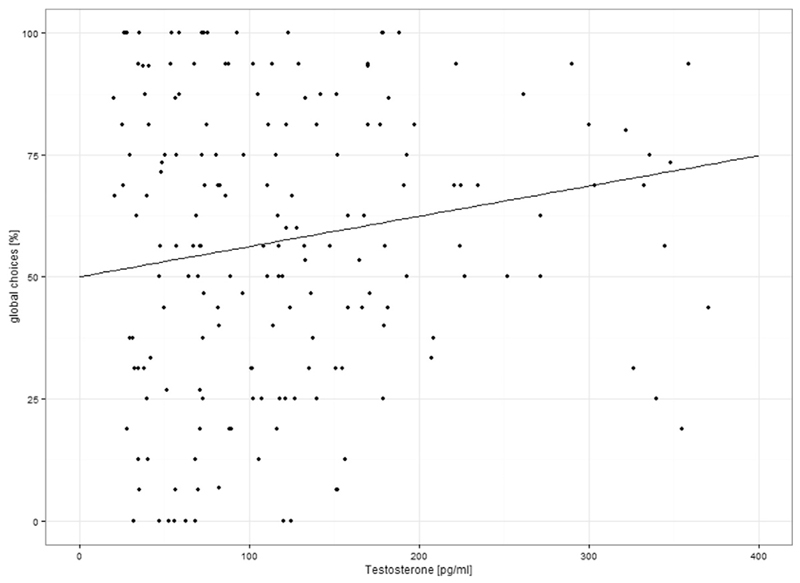
Relationship of testosterone to the percentage of global choices. Irrespective of sex and density, a significant positive relationship was observed between testosterone and the percentage of global choices. The higher the testosterone values the higher was the percentage of global choices.

Irrespective of ‘*density*’, ‘*testosterone*’ had a significant positive influence on the ‘*number of global choices*’ (*b* = 0.64 * 10^-3^, *SE*_b_ = 0.18 * 10^-3^, *t*_(936)_= 3.45, p < 0.001). The higher the testosterone level, the more global choices did participants make (Fig. 4). ‘*Progesterone*’ did not affect the ‘*number of global choices*’.

### 3.5 Sex hormone influences on reaction times associated with global vs. local choices

‘*Testosterone*’ and ‘*progesterone*’ did not relate to reaction times overall and did not interact with the effect of ‘*choice*’ on ‘*RT*’. For both hormones, there were significant three-fold-interactions with ‘*sex*’ and ‘*session*’ (both |b| > 1.89, both *SE*_b_ < 1.69, both t > -2.36, both p < 0.02), which were not analyzed further, since they were not relevant to our research question.

### 3.6 Effects of sex and cycle phase on mood

Neither ‘*positive*’ nor ‘*negative affect*’ were significantly affected by ‘*sex*’ or ‘*session*’ in the total sample or ‘*menstrual cycle phase*’ in women. Irrespective of ‘*sex*’ or ‘*session*’, ‘*testosterone*’ was significantly positively related to ‘*positive affect*’ (*b* = 1.30 * 10^-3^, *SE*_b_= 0.58 * 10^-3^, *t*_(176)_= 2.25, *p* = 0.03), but did not relate to ‘*negative affect*’. ‘*Progesterone*’ was not related to ‘*positive*’ or ‘*negative affect*’ across all participants. ‘*Negative affect*’ and ‘*positive affect*’ were positively related across all participants (*b* = 0.23, *SE*_b_= 0.10, *t*_(182)_= 2.37, *p* = 0.02). Therefore, we also performed the following analyses using the mean of ‘*positive affect*’ and ‘*negative affect*’, a measure that we here term ‘*arousal*’.

### 3.7 Mood influences on the number of global choices

**Fig. 5:**
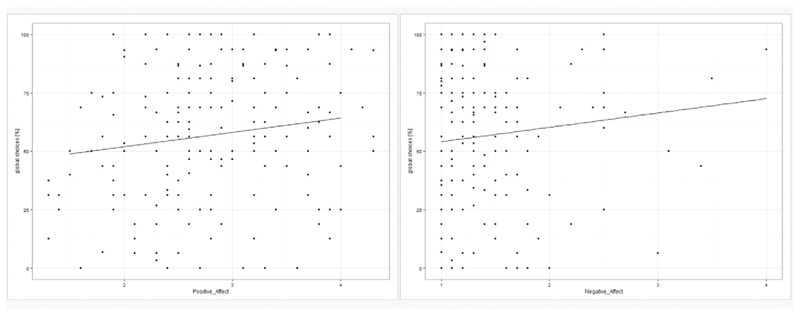
Relationship of positive and negative affect to the percentage of global choices. A significant positive relationship was observed between both, positive and negative affect and the percentage of global choices. The higher the affect the higher were the percentage of global choices.

Irrespective of ‘*density*’, both ‘*positive*’ and ‘*negative affect*’ were significantly positively related to the number of global choices (both *b* = 0.06, both *SE*_b_ < 0.03, both *t*_(967)_> 2.07, both *p* < 0.05, compare Fig. 5). The combined “arousal” measure was also positively related to the ‘*number of global choices*’ (*b* = 0.11, *SE*_b_= 0.03, *t*_(1028)_= 3.15, *p* = 0.002). Since ‘*testosterone*’ was shown to affect both, the ‘*number of global choices*’ and ‘*positive affect*’, a model including both, ‘*positive affect*’ and ‘*testosterone*’ was also tested to address potential mediating effect of ‘*testosterone*’ on ‘*mood*’ or vice versa. Both ‘*testosterone*’ and ‘*positive affect*’ remained as significant factors in the model, but there was no interaction between ‘*testosterone*’ and ‘*positive affect*’.

### 3.8 Mood influences on reaction times associated with global and local choices

‘*Positive affect*’ did not relate to ‘*RT*’ overall, but there was a signifi-cant interaction between ‘*positive affect*’ and ‘*sex*’ (*b* = -456.10, *SE*_b_= 144.14, *t*_(931)_= -3.16, *p* = 0.002). ‘*Positive affect*’ did not interact significantly, albeit negatively, with ‘*choice*’ per se, but there were three-fold interactions between ‘*choice*’, ‘*positive affect*’ and ‘*session*’ (*b* = -275.00, *SE*_b_= 125.92, *t*_(2533)_= -2.18, *p* = 0.03), as well as between ‘*choice*’, ‘*positive affect*’ and ‘*sex*’ (*b* = 383.66, *SE*_b_= 134.23, *t*_(2534)_= 2.86, *p* = 0.004). Therefore, separate models were run for men and women with ‘*menstrual cycle phase*’ included as a factor in women. The results in men demonstrate faster reactions with more ‘*positive affect*’ (*b* = -445.70, *SE*_b_= 113.50, *t*_(768)_= -3.93, *p* < 0.001) as well as a positive interaction between ‘*choice*’ and ‘*positive affect*’ (*b* = 294.0, *SE*_b_= 127.0, *t*_(1745)_= 2.32, *p* = 0.02). This interaction indicates that in men the effect of ‘*choice*’ on ‘*RT*’ was the larger, i.e. processing the more local, the more positive the ‘*mood*’. In women, there was a three-fold interaction between ‘*choice*’, ‘*positive affect*’ and ‘*cycle phase*’ (*b* = 652.0, *SE*_b_= 267.30, *t*_(1307)_= -2.44, *p* = 0.01), resulting from a negative interaction between ‘*choice*’ and ‘*positive affect*’ specifi-cally during the luteal cycle phase. Thus, in women during the luteal cycle phase, the effect of ‘*choice*’ on ‘*RT*’ was the smaller, i.e. processing the more global, the more positive the mood. ‘*Negative affect*’ did not relate to ‘*reaction times*’ overall and did not modulate the effect of ‘*choice*’ on ‘*RT*’. The combined ‘*arousal*’ measure was related to overall ‘*RT*’ (*b* = -203.67, *SE*_b_= 75.31, *t*_(427)_= -2.71, *p* = 0.007), but did not interact with the effect of ‘*choice*’ on ‘*RT*’. Reactions were the faster, the higher the ‘*arousal*’ value.

## 4 Discussion

The primary objective of the present study was to investigate sex differences in global versus local processing utilizing the Kimchi-Palmer task in adults. Previous studies assessing global-local processing with a Navon paradigm [14–15] observed a global processing bias in men, but local processing bias in women. Studies using the Kimchi-Palmer task however, did not observe significant sex differences in the number of global choices in adults [23], even though more global choices in boys than girls had repeatedly been observed in children [21–22]. Since the ability to recognize Gestalt-like global architectures [27–30] develops with age, sex differences in the development of global processing may potentially explain these inconsistencies. The aim of the present study was however, to investigate, whether sex differences in the Kimchi-Palmer task can be replicated in adults, when reaction times instead of the number of global choices are taken into account.

As in the study of Basso and Lowery [23] investigating an adult sample, the current study did not reveal significant differences between adult men and women in the number of global choices. By contrast, analyses concerning reaction times indicate that women's local choices were significantly faster than their global choices and faster than local choices in men. Men on the other hand were slightly faster responding to global matches, compared to women. Against the prominent assumption of an overall global precedence effect (e.g. faster responses to global, compared to local items) [1] we observed overall faster responses to local, compared to global items in men and women in the present sample. Some studies also observe faster local responses in the Navon task [15].

Irrespective of sex, density influenced the number of global choices. Participants decided more often for global comparison figures, when items consisted of more densely packed local elements (e.g. 6 to 9 elements), whereas more local comparison figures were selected when the items consisted of fewer sparsely packed local elements (e.g. 3 to 4 elements). This result is consistent with observations by Martin [5] and Kimchi and Palmer [20], who demonstrate that a low number of local elements (low-density items) encourages local processing, whereas a higher number of local elements (high-density items) facilitates the perception of the global or overall configuration. Notably, reactions times were however not influenced by density in the current study.

Differences in reaction times, but not in the number of global vs. local choices between men and women suggests that sex differences in global-local processing in adults are not so much reflected in global or local choices, but in the time taken to make these choices. There was no significant difference in the number of global choices between men and women, although numerically, men choose global matches more often than women. However, if women decide for global matches they take significantly more time than men to do so.

We speculate that the prolonged reaction times for global choices in women and for local choices in men result from prolonged reflective decision making processes, that may cover the initial response tendency, if participants are not pressed for time. With such prolonged decisions, participants choices may no longer reflect their actual global or local bias. This may also offer a partial explanation on why sex differences in the Kimchi Palmer task were observed in children, but not in adults. It is well-established that children and adolescents are known to be rather impulsive and reward-oriented in decision making or lack self-control. Adults however, exhibit longer and extensive decision making processes, being more reflective and weighing pros and cons carefully before responding to decisions [31–34]. Note however, that impulsivity was not controlled for in the current study. Therefore, future studies following up on this result are warranted to confirm that impulsive decision making modulates sex differences in the number of global and local choices in the Kimchi-Palmer task, for example by including an impulsivity scale or behavioral impulsivity paradigm.

The present investigation did not support the hypothesis that menstrual cycle affects decision making in the Kimchi-Palmer task. While the sex difference in the global processing bias during the Navon paradigm is restricted to the luteal cycle phase [14], we did not find any relation of menstrual cycle phase to performance in the Kimchi-Palmer task, neither with respect to reaction times, nor with respect to the number of global decisions. Accordingly, no relationship of progesterone to either the number or speed of global decisions was observed in the present study. However, the Kimchi-Palmer task may simply not be sensitive enough to detect menstrual cycle dependent differences in global-local processing due to the similarity judgment design, the non-restricted response time and the fact that only 16 items per session are evaluated. These characteristics are important design parameters that differentiate the Kimchi Palmer task from the Navon task.

Nevertheless, sex hormone concentrations seemed to play a role in global-local decision making in the Kimchi-Palmer task in the present investigation. Irrespective of sex, testosterone had a significant positive effect on the number of global choices, i.e. higher testosterone levels were associated with a more global processing bias. This finding is consistent with previous findings on testosterone influences on global reaction times in the Navon paradigm [14]. Neither progesterone, nor testosterone did relate to the effect of choice on reaction times. Thus the difference between global and local reaction times was not influenced by progesterone and testosterone levels.

Consistent to our hypothesis, positive affect was positively related to the number of global choices. However, also negative affect was positively related to the number of global choices. Since the two measures were also positively related in the current sample of psychologically healthy young participants, we assume that this result rather reflects an overall effect of arousal, irrespective of valence. We also confirmed that a measure of “arousal” combining the positive and negative affect values had the same effect on the number of global choices. It has recently been demonstrated that arousal, irrespective of valence, increases signal-to-noise ratio in information processing by drawing attention to more salient stimuli [35]. Since it is usually argued that global forms are more salient than local forms [6] it is possible that a similar process may explain the results of the present study. Importantly, the effect of mood on global choices was not mediated via sex hormone levels. We furthermore demonstrated an effect of positive affect on the effect of choice on RT, which was sex-dependent. Whereas in men positive affect was linked to a local processing bias, in the luteal phase of women, positive affect was related to a global processing bias. Albeit, this has not been tested directly in the present study, it is possible that positive mood lead participants to make even more reflective decisions. Note also that unlike for the number of global choices, negative affect and the combined arousal measure did not elicit similar results.

In summary, with the present investigation we were able to demonstrate sex differences in the Kimchi Palmer task in adults for the first time. By taking into account choice reaction times we were able to demonstrate a stronger local processing bias in women compared to men also in adults.

## Figures and Tables

**Table 1:** Mean levels of testosterone and progesterone in men and women in both cycle phases.

		Men	Women	Women	
		Averaged over both sessions	Averaged over both phases	Follicular	Luteal
Testosterone (pg/ml)	*M*(*SE*)	171.61 (2.35)	74.46 (1.17)	72.77 (1.55)	76.18 (1.75)
Progesterone (pg/ml)	*M*(*SE*)	74.30 (2.22)	241.65 (8.73)	134.47 (8.39)	351.38 (14.26)

Note. *M*= mean; *SE*= Standard error.

## High Lights

- Number of global choices did not differ between adult men and women.
- In women, global choices were slower than local choices.
- Responses to global choices were slightly faster in men, compared to women.
- Testosterone was positively related to the number of global choices.
- Positive and negative affect were both positively related to the number of global choices.
